# Analysing how physical activity competes: a cross-disciplinary application of the Duplication of Behaviour Law

**DOI:** 10.1186/s12966-019-0847-9

**Published:** 2019-12-05

**Authors:** Amy L. Wilson, Cathy Nguyen, Svetlana Bogomolova, Byron Sharp, Timothy Olds

**Affiliations:** 1 Ehrenberg-Bass Institute for Marketing Science, Business School, University of South Australia, Adelaide, South Australia Australia; 2 Alliance for Research in Exercise Nutrition and Activity (ARENA), Sansom Institute, School of Health Sciences, University of South Australia, Adelaide, South Australia Australia

**Keywords:** Physical activity, Competition, Duplication of behaviour, Public health, Marketing

## Abstract

**Background:**

Despite the ongoing promotion of physical activity, the rates of physical inactivity remain high. Drawing on established methods of analysing consumer behaviour, this study seeks to understand how physical activity competes for finite time in a day – how Exercise and Sport compete with other everyday behaviours, and how engagement in physical activity is shared across Exercise and Sport activities. As targeted efforts are common in physical activity intervention and promotion, the existence of segmentation is also explored.

**Methods:**

Time-use recall data (*n* = 2307 adults) is analysed using the Duplication of Behaviour Law, and tested against expected values, to document what proportion of the population that engage in one activity, also engage in another competing activity. Additionally, a Mean Absolute Deviation approach is used to test for segmentation.

**Results:**

The Duplication of Behaviour Law is evident for everyday activities, and Exercise and Sport activities – all activities ‘compete’ with each other, and the prevalence of the competing activity determines the extent of competition. However, some activities compete more or less than expected, suggesting the combinations of activities that should be used or avoided in promotion efforts. Competition between everyday activities is predictable, and there are no specific activities that are sacrificed to engage in Exercise and Sport. How people share their physical activity across different Exercise and Sport activities is less predictable – Males and younger people (under 20 years) are more likely to engage in Exercise and Sport, and those who engage in Exercise and Sport are slightly more likely to Work and Study. High competition between Team Sports and Non-Team Sports suggests strong preferences for sports of different varieties. Finally, gender and age-based segmentation does not exist for Exercise and Sport relative to other everyday activities; however, segmentation does exist for Team Sports, Games, Active Play and Dance.

**Conclusions:**

The Duplication of Behaviour Law demonstrates that population-level patterns of behaviour can yield insight into the competition between different activities, and how engagement in physical activity is shared across different Exercise and Sport activities. Such insights can be used to describe and predict physical activity behaviour and may be used to inform and evaluate promotion and intervention.

## Background

Regular physical activity provides a range of physical, psychological and economic benefits to individuals and to society [1–4]. Despite increased access to health information [5] and substantial physical activity promotion efforts [3, 6], approximately one-third of the global population is still physically *in*active [7–9]. Therefore, understanding physical (in) activity and its promotion is an important area of research and practice [3, 10, 11].

Individuals have a limited amount of time to dedicate towards personal and work-related tasks. Thus, physical activity competes with other activities for individuals’ finite time within a given day, in a 24 h/day sum. The way that people allocate their time across different categories of activities (e.g. Sleep, Leisure, Occupation, Transportation and Home [12]), and the specific behaviours that they choose to engage in (e.g. cycling as a leisure activity or mode of transport), are ongoing “trade-offs” of time and effort, offering the opportunity to be either physically active or inactive. These trade-offs determine the duration and variety of physical activity (both of which contribute to the resulting health benefits [3, 13, 14]), and may differ between males and females, across different age groups [15], and a range of other sociodemographic indicators [6, 16–20]. Therefore, understanding patterns of how different activities compete for finite time within a day should offer insights into the promotion of physical activity. Further, these insights offer a different way of analysing and conceptualising time-use data and offer a method for benchmarking and evaluation of behavioural patterns in time-use epidemiology.

The increasing popularity of cross-disciplinary research suggests that different disciplines offer alternative ways of looking at the same phenomena. Yet, similarities and parallels between disciplines allow us to apply methods and approaches from one discipline to the other to gain a new perspective. It is fruitful to draw parallels between how people allocate their finite time to activities to how they spend their finite money on purchasing products and services in a marketing context. In both scenarios, choices are made (choosing one activity over another, or buying Brand A, rather than Brand B), and a trade-off is required. Further, just as much of people’s spending can be either discretionary (e.g. restaurants, clothes) or non-discretionary (rates, taxes, utilities), so our time use can also be discretionary (sports, gardening) or non-discretionary (sleep, work). Finally, purchases can compete for money both within a category (the Corn Flakes and Cocopops brands compete in the breakfast cereal category), as well as across categories (cereal and bread categories compete for breakfast purchases, as well competing with a mortgage for money). In the same way, physical activity competes across categories of activities (screen time, quiet time, chores) for our time, and within the physical activity category (sport, gym, walking). Given the parallels in people’s choices, trade-offs and finite resources, it is conceivable that different types of activities could compete for time in a similar way to how brands compete for sales. In the same way that purchasing one brand or in one category does not preclude someone from purchasing from a different brand or category on another occasion, engagement in one activity does not preclude someone from engaging in a different activity on another occasion. If this is true, this suggests that the field of marketing science can offer some novel insights into the area of physical activity, for example about how to better promote being active. While activities can indeed be complementary, this paper approaches engagement in activities from the lens of competition – as each activity competes with the others for finite time in the day. Therefore, this paper borrows a method used to assess how brands compete and applies it to analyse how engagement in everyday activities and physical activity can be shared across different activities.

Commercial marketing insights have also been used to progress fields of social importance [21, 22]. In fact, interventions that incorporate marketing principles may be more effective than the application of traditional socio-cognitive theories for influencing physical activity behaviour [23]. However, systematic reviews of physical activity interventions using marketing techniques demonstrate that competitive analysis or analysis of competition – is often overlooked; with inclusion in only 27% of studies reviewed [24, 25]. The importance of competition in non-commercial contexts (e.g. companies, people, and behaviours that influence voluntary behaviour change, such as being physically active) are acknowledged [22, 26], and there is a need for approaches to analyse and understand competition of physical activity behaviour [24, 25].

Scientific laws (empirical generalisations) are advancing in the field of marketing by describing predictable patterns of consumer behaviour [27, 28]. These laws enable marketers to benchmark and predict consumer buying behaviour, and provide insights that carry important practical implications [29]. One particular empirical “law” of marketing science, the Duplication of Purchase Law, describes the likelihood that a randomly chosen individual in a population will purchase any two products in the same category (for example, two different types of breakfast cereal) [30]. In this study, we explore whether a behavioural analogue of the Duplication of Purchase Law, the Duplication of *Behaviour* Law, is evident at both the category (competing types of everyday activities such as Work and Study, quiet time, physical activity) and physical activity “brand” (competing types of physical activity, such as gym, sport, walking) level. We report an empirical study that tests whether the Duplication of Behaviour Law can predict the likelihood of a randomly chosen individual participating in any combination of everyday and physical activities. Finally, we discuss the implications of this competition for marketing physical activity.

### Duplication of Purchase (Behaviour) Law

The Duplication of Purchase Law [31] is an empirical description of how brands share customers, that is, how they compete for customers. The Law has been extensively tested across a range of purchasing contexts (consumer packaged goods [32, 33], fruit and vegetables [34] and sports brands [35]), and non-purchase-related choice behaviours (TV viewing behaviour [36], listening to the radio [37], cultural event attendance [38], gaming [39], gambling [40], leisure time activities [41], sport attendance and team preferences [42], and physical activity [43]). The application of the Law across such a wide variety of conditions demonstrates its robustness, and superior ability to explain and predict competition for different types of consumer behaviours.

To match the physical activity context of this paper, we propose that this pattern is referred to as the Duplication of *Behaviour* Law, and would suggest that for physical activity:

1. People have “polygamous” loyalty – they share their engagement across multiple alternatives (not just one single option), and that similar behaviours may co-occur or compete with each other; and
2. The probability of participants engaging in various combinations of daily activities, and physical activities, can be predicted quantitatively based on the population prevalence of the constituent behaviours. If the Duplication of Behaviour Law holds for physical activity, visual inspection of the pattern and the expected values of the Dirichlet model enable reliable prediction of how many people *both* go to the Gym *and* play Sport, if the number of people who go to the Gym and how many play Sport is known. If the Law does not hold, it may be evidence of a boundary condition, but would require further replication to confirm this.

As with classic empirical laws (such as the laws of physics), the Duplication of Behaviour Law allows a straightforward analysis that is easy to use, interpret and replicate, carrying important implications for research and industry [44, 45].

### Theoretical applicability to exercise behaviour

Probability theory underpins the Duplication of Purchase Law [31, 46]. An assumption of the Law, relevant to the physical activity context of this study, is that the population has minimal segmentation [46]. That is, participation in any given activity is not dependent on some socio-demographic characteristic, such as age or gender. It is possible that physical activity behaviour might violate this assumption, given that different groups of people, also called “segments” (defined based on socio-demographic and psychographic variables such as age and gender [15]), have been shown to engage in physical activity differently (6, 16, 17, 18 l, 19, 20). However, inconsistencies in the sociodemographic make-up of these segments [6, 16, 17] and their instability over time [47] have been noted. Inconsistencies may be attributed to different analytical approaches [48] or, more controversially, may indicate the absence of segments meaningful enough for targeted marketing intervention [49, 50].

Instead, the Mean Absolute Deviation (MAD) analysis is another approach to segmentation analysis [49] and is advantageous as it eliminates potential outlier bias [51, 52]. It is also a simple method of analysis that can be readily replicated, and provides an output that can be compared across studies – allowing for stability or instability of segments across studies and over time to be detected. The MAD analysis has found that the demographic characteristics of users of competing brands seldom differ [53, 54], even over time [55], thus suggesting minimal segmentation. As MAD analysis is an appropriate approach to test for segmentation, it will be used to test for segmentation in the present study.

### Applying the Duplication of Behaviour Law to physical activity

Duplication analysis underpins the Duplication of Behaviour Law and assesses the competition between alternatives. In the context of this study, duplication analysis offers a method of understanding how people exchange their time across activities and therefore how different activities co-occur. To the best of our knowledge, there is currently no documented evidence of the Duplication of Behaviour Law applied to everyday activities. Therefore, the first objective of this paper is to investigate how physical activity competes with other activities (e.g. sleep, chores, screen time etc.) for people’s time in a day.

The second objective of this paper is to investigate how engagement in physical activity is shared across different types of physical activities (e.g. gym, team sports). Only one study has looked at Duplication of Behaviour Law in the context of physical activity [43]. The study found that the competition for specific activities (e.g. walking, football etc.) was very low, with most combinations of activity undertaken by less than 0.1% of the sample. Low competition could be due to the very fine granulation of physical activities where the time period and sample sizes were too small to apply the duplication analysis appropriately. As a result, little could be inferred other than the general qualitative pattern of the Duplication of Behaviour Law; that is participants in any one activity were more likely to participate in another activity if it was more popular among the total population.

Finally, the demographic similarities or differences of engagers are often a consideration in the development of physical activity interventions and promotion. Therefore, the third objective is to test for segmentation with the Mean Absolute Deviation approach [49, 53].

## Method

### Sample and data

Data were obtained from 17 studies of adults across Australia and New Zealand. Of the 17 studies, 10 were cross-sectional studies, five were randomised controlled trials, one cohort study and one pre-post study. Of all the studies, convenience samples were primarily used. In total, they covered 2307 adults, 56% of whom were females, at various stages of life (e.g. university students, shift workers and retirees). Ages ranged from 16 to 96 years, with an average age of 33 years.

Data in all studies were collected using the same protocols and analytical procedures. The data were collected using the Multimedia Activity Recall for Children and Adolescents (MARCA); a 24-h recall method using the day reconstruction technique and a segmented-day format. Participants recalled an average of 3.1 days through computer-assisted telephone interviews. Originally designed to capture children’s physical activity behaviours [56], the MARCA has very strong same-day test-retest reliability (0.98–1.00), and moderate to strong validity when compared with results from accelerometry and the gold standard doubly-labelled water method [57]. These results show that MARCA is a feasible and accurate measure of adults’ physical activity behaviour and energy expenditure [58]. The data captured all activities engaged in in a 24 h period and were weighted 5:2 for weekdays:weekend days. Participants choose from 520 different activities (e.g. lifting weights, eating, reading), which were then organised into 11 activity domains: Sleep, Self-care (e.g. grooming, showering), Social (e.g. talking to or message friends), Cultural (e.g. art), Screen Time (e.g. video games, phone), Chores (e.g. house cleaning, laundry), Work and Study, Quiet Time (e.g. reading), Active Transport (e.g. cycling, walking), Passive Transport (e.g. car, bus) and Exercise and Sport. The Exercise and Sport domain was categorised into six sub-domains: Non-Team Sports, Team Sports, Gym, Dance, Active Play, and Games. While Active Transport includes physically active behaviour, this domain comprised primarily of walking (95% of total Active Transport time). For most individuals, walking is required to move around in everyday life, and often at a very low intensity, therefore Active Transport was not included in the physical activity level analysis, leaving Exercise and Sport as the representation of physical activity.

### Analysis

First, the prevalence of each activity/physical activity was calculated, which is the number of people who engaged in an activity divided by the total sample size, for both the activity domain and Exercise and Sport domain.

Next, duplication (i.e. sharing) values were calculated, being the proportion of people who engaged in one activity who *also* engaged in another (in the time period of study). These figures were presented in a duplication table (see Table 1) and sorted by the prevalence of each activity (highest to lowest) in both the columns, and the rows. The fit of the Duplication of Behaviour Law is evident through visual analysis, where the expected patterns should “stand out very clearly to the naked eye” (Ehrenberg 1988; p. 193). For interpretation, the table is read from left to right (see Table 1). Duplication values that decrease across rows and down columns, also demonstrated by averages, indicate the Duplication of Behaviour Law.

**Table 1:** Duplication of behaviour for the activity domain

		% of people who also engage in …										
Activity	Prevalence (%)	Sleep	Self-Care	Social	Passive Trans	Screen Time	Chores	Work & Study	Quiet Time	Active Trans	Exercise & Sport	Cultural
Sleep	100		100	94	93	92	91	88	87	86	55	14
Self-Care	100	100		94	93	92	91	88	87	86	55	14
Social	94	100	100		95	92	92	89	89	88	55	14
Passive Transport	93	100	100	95		93	92	89	88	87	55	14
Screen Time	92	100	100	94	94		92	88	88	87	55	13
Chores	91	100	100	95	94	93		88	89	87	54	14
Work & Study	88	100	100	95	95	92	92		89	88	58	14
Quiet Time	87	100	100	95	94	93	93	90		89	55	15
Active Transport	86	100	100	96	95	93	92	90	90		56	14
Exercise & Sport	55	100	100	95	95	93	90	93	89	89		15
Cultural	14	100	100	97	94	89	94	89	94	89	58	
Average		100	100	95	94	92	92	89	89	87	56	14
Expected		100	100	95	94	93	92	89	88	87	55	14
Correlation	0.99											

The Duplication coefficient (D) provides an overall assessment of the prevalence of competition [59], indicating the population likelihood that someone who engages in one behaviour also engages in another. It is calculated as the average duplication divided by the average population prevalence of participation in a given activity across all activity categories. D > 1 shows that engaging in one activity means someone is more likely than the population average to engage in another activity. D < 1 suggests that engaging in one activity is associated with lower than the population likelihood of engaging in another.

D is then used to calculate theoretical (expected) values – D multiplied by individual penetration values. The expected values are then compared to the observed values (average duplication figures) to determine the fit of the Duplication of Behaviour Law numerically [34].

Finally, deviation values were calculated by subtracting the observed duplication values from the average duplication figures (for each column/behaviour). Deviations for everyday activities are presented in Table 2. A positive deviation suggests that co-occurrence of one activity with another is higher than expected, whereas a negative value indicates less co-occurrence than expected. A deviation of zero indicates that the rate of co-occurrence is as would be expected, based on the expected values. There are two ways of interpreting these figures. Deviations that are at least ±5 percentage points (pp) from the average were considered to reflect practically important differences, consistent with prior research investigating Duplication of Behaviour for leisure time activities [41]. Deviations that occur in both directions (e.g. sport co-occurs more with gym *and* gym co-occurs more with sport) are known as “partitions”. Partitions signal when two behaviours co-occur at a higher or lower level than would be expected for that population. Partitions are based on the respective penetration of the activities and the overall duplication of activities.

**Table 2:** Deviations for everyday behaviours

		% of people who also engage in …										
Activity	Prevalence (%)	Sleep	Self-Care	Social	Passive Trans	Screen Time	Chores	Work & Study	Quiet Time	Active Trans	Exercise & Sport	Cultural
Sleep	100		0	−1	− 1	0	− 1	− 1	−2	− 1	− 1	0
Self-Care	100	0		−1	− 1	0	− 1	− 1	−2	− 1	− 1	0
Social	94	0	0		1	0	0	0	0	0	−1	0
Passive Transport	93	0	0	0		0	0	0	−1	0	0	0
Screen Time	92	0	0	−1	0		0	− 1	− 1	− 1	−1	− 1
Chores	91	0	0	0	0	1		−1	0	0	−2	0
Work & Study	88	0	0	0	0	0	0		0	0	2	0
Quiet Time	87	0	0	0	0	1	1	1		1	0	1
Active Transport	86	0	0	1	1	1	0	1	1		1	0
Exercise & Sport	55	0	0	0	1	0	−2	4^a^	0	1		1
Cultural	14	0	0	2	0	−3	2	0	**5**	1	3	
Average	82	100	100	95	94	92	92	89	89	87	56	14
D-Coefficient	1.0											
MAD	0.6											

Bolded value = deviation of ± 5 pp

^a^ *=* Slight deviation, just short of the ± 5 pp. cut off

Next, a user profile analysis [49, 60] was conducted to investigate whether segmentation exists for activities (every day and types of physical activities), consistent with Scriven et al. [41]. The demographic composition (based on gender and age) of people that engage in different activities is compared with the average proportion across all activities. Deviations above or below five percentage points from the average are highlighted, as this may indicate a meaningful demographic difference [50]. A Mean Absolute Deviation (MAD) figure is also calculated as the average absolute deviation across all activities, to indicate the demographic variability across competing alternatives. A lower MAD indicates less segmentation. A MAD below five, while arbitrary, is unlikely to be practically significant [49, 55] – that is, the differences between these groups are insufficient enough to warrant tailored campaigns for each of the segments, as opposed to a more mass-appeal campaign that can reach more people.

## Results

### Objective one: how Exercise and Sport competes with other behaviours for consumers’ time

The number of activities that people engaged in ranged from three to 11, with a median of nine and an interquartile range of two. On any one day, three-quarters of the sample engaged in nine or more activities. Thus, time is shared across a wide range of everyday activities, and supports that there is extensive competition for time.

A Spearman Rank correlation of 1.00 between observed and expected values indicates a good fit of the Duplication of Behaviour Law for everyday activities (Table 1). All activities compete with each other – that is, for every combination of two activities, at least one person engages in both. Exercise and Sport compete for time with all other activities, with the degree of competition determined by the prevalence of the competing activity. For example, Exercise and Sport compete more with Socialisation and Passive Transport, Screen Time, Chores, and Work and Study, with more than 90% of people who engage in physical activity also engaging in these other activities. However, Exercise and Sport compete less with less prevalent activities such as Cultural Activities, with only 14% of people who are physically active also engaging in Cultural Activities. For everyday activities, D = 1.0, signalling that across the whole domain, engaging in one activity makes a person no more or less likely to engage in any of the other activities. This is likely a result of almost everyone engaging in these activity domains. A MAD of 0.6 indicates few deviations from competition as predicted by the model. Table 2 shows that at the ±5 pp. deviation threshold (bolded figure), people who engage in Cultural Activities are slightly more likely to also engage in Quiet Time (e.g. reading). However, this may be due to the low prevalence of Cultural Activities. There is a slight tendency for people who engage in Exercise and Sport to also Work and Study (4 pp. deviation-indicated with an asterix). No partitions are evident.

Table 3 presents the gender composition for everyday activities. The average deviation across all activities is very low, indicating a lack of gender-based segmentation for everyday activities. For example, of the 1697 people who use Active Transport, 43% of them are males, and 57% of them are females; close to the population gender split of 56% females and 44% males. While all deviations from the average are under five percentage points, there is a slight skew towards females for Cultural Activities (− 4 pp), and towards men for Exercise and Sport (+ 4 pp), compared to the average.

**Table 3:** Gender composition of everyday activities– proportions and deviations

		Gender			
		Proportion of engagers (%)		Deviation from average	
	n	Male	Female	Male	Female
% sample (*n* = 2307)		44	56	44	56
Sleep	2307	44	56	0	0
Self-Care	2306	44	56	0	0
Social	2167	43	57	−1	1
Passive Transport	2154	44	56	0	0
Screen Time	2125	44	56	0	0
Chores	2107	42	58	−2	2
Work & Study	2026	44	56	0	0
Quiet Time	2011	43	57	−1	1
Active Transport	1984	43	57	−1	1
Exercise & Sport	1261	48	52	4^a^	−4
Cultural	313	40	60	−4	4^a^
Average		44	56	MAD = 1	

^a^ *=* Slight deviation, just short of the ± 5 pp. cut off

Table 4 presents the composition for everyday activities across four age groups. The average deviation across most of the activities is low, with a slight skew towards younger people (20 years and under) for Exercise and Sport. The only activity with a skew that exceeds the ±5 deviation is Cultural Activities, which skews towards older adults aged 60 or more (+ 8 pp), and away from young adults between 20 and 40 years (− 6 pp).

**Table 4:** Age composition of everyday activities– proportions and deviations

		Age (years)							
		Proportion of engagers (%)				Deviation from average			
Activity	n	< 20	20–39	40–59	60+	< 20	20–39	40–59	60+
% of sample (*n* = 2307)		33	40	10	17	33	40	10	17
Sleep	2307	31	41	11	17	0	1	0	−1
Self-Care	2306	32	41	11	17	0	1	0	−1
Social	2167	31	40	11	18	0	0	0	−1
Passive Transport	2154	31	41	11	17	0	1	0	−1
Screen Time	2125	31	40	11	18	0	0	0	0
Chores	2107	29	41	12	18	−2	1	1	0
Work & Study	2026	32	41	11	15	1	1	0	−3
Quiet Time	2011	30	39	12	19	−1	−1	1	1
Active Transport	1984	31	39	12	18	−1	− 1	1	0
Exercise & Sport	1261	35	41	9	16	4^a^	1	−2	−2
Cultural	313	30	34	9	27	−1	**−6**	−2	**8**
Average		31	40	11	18	MAD = 1			

Bolded value = deviation of ± 5 pp

^a^= Slight deviation, just short of the ± 5 pp. cut off

### Objective two: how engagement in physical activity in shared across Exercise and Sport activities

Of the 1261 people who engaged in Exercise and Sport, the number of activities engaged in ranged from one to five (out of six), with a median of one and an interquartile range of one. The sharing of physical activity across Exercise and Sport activities follows the Duplication of Purchase Law, such that it varies in line with the relative prevalence of each of the activities (see Table 5). For example, of the 41% of people who play Non-Team Sports, 23% also play Team Sports (a more prevalent activity), and only 6% Dance (a less prevalent activity). The average duplication figures decrease from left to right. The Spearman Rank correlation between the average and expected values is 1.00, indicating that the degree of sharing can be predicted based on the prevalence of the other activity.

**Table 5:** Duplication for Exercise and Sport activities

		% of people who also engage in…					
Activity	Prevalence % *n* = 1261	Gym	Non-Team Sports	Team Sports	Active Play	Games	Dance
Gym	60		34	19	6	4	5
Non-Team Sports	41	49		23	7	5	6
Team Sports	14	46	38		5	8	3
Active Play	10	38	28	13		6	5
Games	7	32	30	27	8		4
Dance	7	43	36	12	7	5	
Average	23	42	33	19	7	5	5
Expected		48	32	11	8	6	6
Correlation	1.00						

Overall, there is a slight departure from the model norms, with a MAD of 2.5. Table 6 shows that there are some deviations at the ±5% threshold (bolded values). Compared to the expected values, people who play Non-Team Sports are more likely to go to the Gym, people who engage in Active Play (e.g. playing with pets) are less likely to play Sport (team or non-team), and people who play Games are less likely to go to the Gym and more likely to play Team Sports.

**Table 6:** Deviations for Exercise and Sport activities

		% of people who also engage in…					
Activity	Prevalence % n=1261	Gym	Non-Team Sports	Team Sports	Active Play	Games	Dance
Gym	60		1	0	0	-2	0
Non-Team Sports	41	**8**		**5**^a^	0	0	1
Team Sports	14	4	**5**^a^		-2	2	-1
Active Play	10	-4	**-5**	**-6**		0	0
Games	7	**-9**	-3	**8**	1		0
Dance	7	2	3	**-7**	1	-1	
Average	23	42	33	19	7	5	5
D-Coefficient	0.8						
MAD	2.7						

Bolded value = deviation of ±5 pp

^a^partition

There is also one partition (boxed cells), where those who play Team Sports are more likely to play Non-Team Sports, and vice versa. People share their time across Sports, and Non-Team Sports more than expected.

The D-coefficient of 0.79 suggests that people who engage in one activity have a 21% decreased chance of engaging in another, relative to the population likelihood as predicted by the model. So, engaging in one Exercise and Sport activity decreases your chance of engaging in any other.

Table 7 presents the gender composition for Exercise and Sport activities. Overall, there is a MAD of 12, indicating a high degree of gender-based segmentation for Exercise and Sport activities. Bold values indicate deviations ±5 from the average. There are skews towards males for Team Sports and Games (e.g. Frisbee). Whereas, there are skews towards women for Active Play (e.g. playing with pets, playing catch) and Dance. However, Gym and Non-Team Sports have no gender segmentation.

**Table 7:** Gender composition for Exercise and Sport activities – proportions and deviations

		Gender			
		Proportion of engagers (%)		Deviation from average	
	n	Male	Female	Male	Female
% sample (*n* = 2238)		44	56	44	56
Gym	754	48	52	−1	1
Non-Team Sports	514	48	52	−1	− 1
Team Sports	317	63	37	**14**	**−16**
Active Play	127	35	65	**−14**	**12**
Games	90	67	34	**18**	**−19**
Dance	83	24	76	**−25**	**23**
Average		49	53	MAD = 12	

Bolded value = deviation of ± 5 pp

Some Exercise and Sport activities show variation across age groups (Table 8), but to a far lesser extent than for gender, with MAD of 4.4. Team Sports and Games skew towards younger people (under 20 years of age), and away from older people (60+ years). Whereas, Active Play is engaged in by fewer people aged 20 and under, and more by those aged 40 and over. There is also a slight skew away from under 20-year-olds for Gym. Non-Team Sports and Dance have similar age profiles.

**Table 8:** Age composition of Exercise and Sport activities– proportions and deviations

		Age (years)							
		Proportion of engagers (%)				Deviation from average			
	n	< 20	20–40	40–60	60+	< 20	20–40	40–60	60+
% of sample (*n* = 2238)		33	40	10	17	33	40	10	17
Gym	754	33	41	8	18	**−5**	1	1	4
Non-Team Sports	514	36	38	11	15	−2	−2	4	1
Team Sports	317	53	40	3	3	**15**	0	−4	**−11**
Active Play	127	26	37	13	24	**−12**	−3	**6**	**10**
Games	90	46	43	2	9	**8**	3	**−5**	**−5**
Dance	83	36	42	7	14	−2	2	0	0
Average		38	40	7	14	MAD = 4.4			

Bolded value = deviation of ± 5 pp

## Discussion

### Everyday activities compete similarly, and this competition is predictable

Exercise and Sport compete with other everyday activities, and leisure-time activities [41] in line with the Duplication of Behaviour Law. Importantly, all discretionary behaviours compete in a predictable way. People who do any one activity (e.g. Chores) are more likely to also engage in activities that are more prevalent (e.g. Socialising) and less likely to spend time engaging in less prevalent activities (e.g. Exercise and Sport). Thus, physical activity has similarities with other consumer behaviours. However, it is important to note that the current paper sought to understand competition for time within a day and as such, had to include all activities that make up a 24-h period. Naturally, this resulted in the inclusion of activities which are highly prevalent (Sleep, Self-care). While the large degree of duplication means that the fit of the law is unsurprising, the evidence of some deviations for the less prevalent activities, including physical activity, demonstrate how different physical activity is and why it warrants further investigation.

### Exercise and Sport is not achieved by sacrificing other behaviours

The similar pattern of competition across all activities suggests that the allocation of time to Exercise and Sport is not at a substantial sacrifice of any other particular activity – but rather, slightly less allocation of time across activities, also evidenced in prior research [41].

### People who do Exercise and Sport are slightly more likely to Work and Study

While the somewhat higher competition between Exercise and Sport, and Work and Study is not substantial enough to warrant specific intervention, it would be interesting to explore why this may occur.

### Males and those under 20 years are more likely to engage in Exercise and Sport

Results from the segmentation analysis suggest that everyday activity domains are largely unsegmented and therefore the messages, creative and media selection used in the promotion of Exercise and Sport (or any other everyday activity) should reach and be relevant to men and women of all ages. This aligns with the population-level approach to preventative health – that engaging more people rather than targeting specific groups can have a greater population-level impact through societal and shifts in behaviour [61].

The slight skew towards males and younger people for Exercise and Sport is consistent with prior research [3, 41]. Yet, the MAD analysis suggests that these differences are not sufficient enough to warrant targeted marketing intervention. Given that gender inequality in sport has been topical in the literature, further research is required [62–64].

### Patterns of engagement in Exercise and Sport activities is less predictable

The Duplication of Behaviour Law is also evident for Exercise and Sport activities. However, overall there is minimal sharing of physical activity across these activities. Less than half of those who engage in Exercise and Sport engage in more than one. In fact, playing one sport decreases your chance of playing any other.

### Higher or lower sharing between activities reveals insights about people’s preferences

For those who do engage in more than one Exercise and Sport activity, the deviations from the Law do offer insights into what might drive people to choose different combinations of activities [41]. For example, the finding that people who play Non-Team Sports are more likely to go to the Gym, and those who Dance are less likely to play Team Sports suggests that some people have preferences for solo activities, consistent with prior research [41]. In contrast, people who play Games are more likely to play sports and less likely to go to the Gym or play Non-Team Sports, which suggests a preference for group activities. The finding that people who engage in Active Play (e.g. playing with animals) are less likely to engage in Gym, Non-Team Sports and Team Sports suggesting that pets may be a motivation for activity, rather than the physical activity itself. The partition between Team Sports and Non-Team Sports suggests a strong preference for sport-related activities. As a variety of activities can enhance health benefits [3, 4, 14], the deviations can inform suggestions about which combinations of activities to promote together or separately to encourage engagement for those who are not already active, and increased variety for those who are. It may also inform the development of content and communications that appeal to people’s preferences, but this requires further investigation.

### Several Exercise and Sport activities exhibit segmentation

Segmentation exists for Team Sports, Games, and Active Play and Dance. These insights have greater relevance to organisations and clubs that affiliate with particular activities (e.g. sporting clubs, Dance studios, Gyms). As demographic information including age and gender can be used by marketers to determine the target audience and guide media selection [65], these insights can inform how and where specific Exercise and Sport activities should be promoted.

### Limitations and future research

As the Duplication of Behaviour analysis considers the substitutability of alternatives, it may be that people do not perceive different sports as alternatives (i.e. not directly substitutable), and hence sharing of people is less evident. Alternatively, it may be that the three-day period is not a long enough time frame to adequately detect the full extent of patterns and combinations of Exercise and Sport activities. Further, as engagement is only treated as a dichotomous variable, the amount of time spent engaging in any one activity was not considered. Therefore, future research should consider a longer time period and an analysis that considers competition with consideration for amount of time spent engaging in each activity.

The level of granulation of activities influences the results. At a low level of granulation (fewer domains, as used in this study), there are high rates of engagement with most activities, allowing for the Duplication of Behaviour Law to emerge. Whereas, at a low level of granulation (i.e. higher number of domains), the Duplication of Behaviour Law is less likely to apply because very few people engage in each activity, as demonstrated in prior research [43]. Any future applications of the Law need to consider the hierarchy and aggregation of behaviours/activities to ensure meaningful results can be drawn out.

In the nature of the marketing science approach and empirical generalisation research, this study sought to assess whether the Duplication of Behaviour pattern occurs in a physical activity context, describing the sharing of time across different everyday and physical activities. While the present study and prior research [43] confirms that the pattern occurs for physical activity, the varied measures, time periods, activities and contexts and socio-demographic factors relevant to physical activity and the competing everyday behaviours suggests the need for further replication and extension to determine the generalisability of the pattern to physical activity under different conditions. While the present study uses a large and diverse sample of adults from Australia and New Zealand, the analysis requires replication of this research across many sets of data (including different samples, time periods, countries and contexts). Replications would add greater validity to the results, consistent with an empirical generalisation research approach [27, 66, 67].

The Duplication of Behaviour Law offers two advantages beyond the previous use of probability theory when it comes to analysing competition of behaviours. Firstly, it has established analysis for determining predicted or expected values that the observed values can be compared against. Second, the extensive replication and extension across many commercial and non-commercial settings, all with similar findings, gives greater confidence in both the results and interpretation, as well as informing the practical implications. Finally, while potential confounding variables are not specifically noted, the descriptive nature of the model means that it does not require confounders as inputs for the analysis. Confounders are built into the model such that the generalisable patterns are evident, independent of these influences. The variations at an individual level are not required for understanding population-level behaviour. However, deviations to the pattern may suggest that there are some confounding variables of interest, in which case, future research may use a different research approach to explore these variables and their influence on behaviour.

Despite the limitations, the results provide a foundation for future research.

Firstly, sharing time across activities means that in order to change behaviour, time must be taken away from one activity and relocated to another. While the extent of this re-allocation cannot be determined through this paper due to the use of dichotomous variables, previous research found that when people start exercising, they take time away from sleeping, watching TV and doing chores, and when they stop exercise they give the time back to those domains [68, 69]. However, this is not a direct transaction of time. For example, they may take 15 min from sleep, reallocate that to chores and then take 15 min from chores and designate that to exercise [68]. The findings suggest that promotion should encourage people to borrow bits of time from other activities to free up the time to allocate to physical activity. Yet, the health, psychological and social effects of these reallocations should be considered as reallocation from TV time, or passive transport to physical activity is likely to have better outcomes than reallocation of sleep time to physical activity. Future research should consider time spent engaging in activities to further explore these trade-offs of time and give a more comprehensive view of how different combinations of activities take up finite time in the day.

Second, the excessive competition between some activities (such as Work and Study) may imply that some people have better time management, sedentary occupations encourage people to allocate some of their time to Exercise and Sport, and/or those who study and work are non-retirees and therefore are still active. Further research is warranted to understand whether this occurrence is evident across additional samples, and if so, what might be the driver of this competition.

Finally, segmentation analysis may be used to identify those who are already engaging in activities, so that promotions can be targeted towards them, or to investigate those who are not engaging and explore ways to make the activity more appealing to a wider audience. To non-engager, this may have a greater chance of growing the overall rates of Sport or Exercise, in the same way that increasing the customer base (and not loyalty of the existing customer base) is the route to brand growth in commercial marketing [44, 70]. Deviations may also inform the existence of groups of people who have a particularly strong preference for or non-engagement in pairs of activities. However, the consistency of segments and deviations over studies and time, consideration of other demographic variables, and the route to physical activity growth requires more research before conclusive insights can be made.

## Conclusions

This study draws parallels between the health and marketing disciplines, adding to both the health and marketing literature by expanding the Duplication of Behaviour Law to investigate physical activity. Application of the Duplication of Behaviour Law to everyday activities and Exercise and Sport activities provides a lens to understand competition for physical activity, and how engagement in physical activity is shared across different Exercise and Sport activities. This study demonstrates that the Duplication of Behaviour Law can be used to (1) analyse population-level competition between behaviours, (2) provide preliminary insights into which combinations of activities should be promoted together (or separately), and (3) enable benchmarking and prediction for researchers and practitioners to evaluate physical activity interventions and promotion. The findings suggest that physical activity has similarities with other consumer behaviours – the prevalence of activities determine the extent of competition between them. Further, the promotion of overall Exercise and Sport activity should consider the promotion of combinations of activities, appeal to people’s preferences, and reach a wide audience. The promotion of specific Exercise and Sport activities may benefit from tailored efforts.

## Data Availability

The datasets used and/or analysed during the current study are available from the corresponding author on reasonable request.

## Abbreviations

MAD: Mean Absolute Deviation
